# Final Opportunity to Rehabilitate an Urban River as a Water Source for Mexico City

**DOI:** 10.1371/journal.pone.0102081

**Published:** 2014-07-23

**Authors:** Marisa Mazari-Hiriart, Gustavo Pérez-Ortiz, María Teresa Orta-Ledesma, Felipe Armas-Vargas, Marco A. Tapia, Rosa Solano-Ortiz, Miguel A. Silva, Isaura Yañez-Noguez, Yolanda López-Vidal, Carlos Díaz-Ávalos

**Affiliations:** 1 Laboratorio Nacional de Ciencias de la Sostenibilidad, Departamento de Ecología de la Biodiversidad, Instituto de Ecología, Universidad Nacional Autónoma de México, México, D.F., México; 2 Departamento de Biología, Facultad de Ciencias, Universidad Nacional Autónoma de México, México, D.F., México; 3 Coordinación de Ingeniería Ambiental, Instituto de Ingeniería, Universidad Nacional Autónoma de México, México, D.F., México; 4 Posgrado en Ciencias de la Tierra, Instituto de Geología, Universidad Nacional Autónoma de México, México, D.F., México; 5 Posgrado en Ciencias Biológicas, Facultad de Ciencias-Instituto de Ecología, Universidad Nacional Autónoma de México, México, D.F., México; 6 Posgrado de Ciencias Bioquímicas, Departamento de Ingeniería Celular y Biocatálisis, Instituto de Biotecnología, Universidad Nacional Autónoma de México, Cuernavaca, Morelos, México; 7 Programa de Inmunología Molecular Microbiana, Departamento de Microbiología y Parasitología, Facultad de Medicina, Universidad Nacional Autónoma de México, México, D.F., México; 8 Departamento de Probabilidad y Estadística, Instituto de Investigaciones en Matemáticas Aplicadas y en Sistemas, Universidad Nacional Autónoma de México, México, D.F., México; University of Waikato (National Institute of Water and Atmospheric Research), New Zealand

## Abstract

The aim of this study was to evaluate the amount and quality of water in the Magdalena-Eslava river system and to propose alternatives for sustainable water use. The system is the last urban river in the vicinity of Mexico City that supplies surface water to the urban area. Historical flow data were analyzed (1973–2010), along with the physicochemical and bacteriological attributes, documenting the evolution of these variables over the course of five years (2008–2012) in both dry and rainy seasons. The analyses show that the flow regime has been significantly altered. The physicochemical variables show significant differences between the natural area, where the river originates, and the urban area, where the river receives untreated wastewater. Nutrient and conductivity concentrations in the river were equivalent to domestic wastewater. Fecal pollution indicators and various pathogens were present in elevated densities, demonstrating a threat to the population living near the river. Estimates of the value of the water lost as a result of mixing clean and contaminated water are presented. This urban river should be rehabilitated as a sustainability practice, and if possible, these efforts should be replicated in other areas. Because of the public health issues and in view of the population exposure where the river flows through the city, the river should be improved aesthetically and should be treated to allow its ecosystem services to recover. This river represents an iconic case for Mexico City because it connects the natural and urban areas in a socio-ecological system that can potentially provide clean water for human consumption. Contaminated water could be treated and reused for irrigation in one of the green areas of the city. Wastewater treatment plants and the operation of the existing purification plants are urgent priorities that could lead to better, more sustainable water use practices in Mexico City.

## Introduction

In megacities with populations exceeding ten million inhabitants, water is a scarce resource that is in high demand. The exploration and application of new, more sustainable water use and reuse approaches are urgently needed. Water managers must change the perspectives and policies that assume that water can only be used once by considering the benefits obtained from the natural environment surrounding the urban areas.

In emerging economies, cities grow before sufficient hydraulic infrastructure has been developed to handle the water supply and wastewater disposal requirements [1]. This lack of infrastructure significantly affects the surface and groundwater systems, making waterways vulnerable to pollution and leading to deleterious effects on water sources and human health. There is a need for more adequate approaches regarding processes that occur at the basin level, in addition to directly considering hydrological ecosystem services, such as water provision and water quality.

During the evolution of what is now the Mexico City Metropolitan Area (MCMA), lakes and rivers have been transformed into drains or converted into sewers in the vicinity of modern avenues, and freeways transit over piped waterways [2]. The hydraulic system of the Basin of Mexico, where the MCMA and its 22 million inhabitants are located, has been irreversibly altered. Of the 45 rivers in this region, most are piped to avoid flooding and unsanitary conditions.

In the Basin of Mexico, the current water demand is 77 m^3^/s for first use, of which 71% is groundwater extracted through wells, 2% is derived from springs and surface water, 21% is from the Cutzamala surface water system, and 6% is from the Lerma groundwater system [3], . The subject of this research, the Magdalena-Eslava river system, represents the most important surface water source for human use in Mexico City.

Rivers flow from their origins in high areas to low-lying areas, exposing these water bodies to the integrated effects of human activities along their course and throughout the basin landscape. Anthropogenic impacts have impaired the ability of many river ecosystems to provide the goods and services upon which society depends. Watercourses may be degraded and rendered unable to support healthy aquatic communities such that natural processes are hampered and social value is lost.

Balancing the interactions between natural and constructed systems in urban areas is crucial for the future supply of water for large human settlements. Interest in river rehabilitation and restoration exists at the global level. River restoration is defined as an improvement in the integrity of a river through human intervention. The aim of river restoration is either to recover the hydrologic and ecological processes or to achieve rehabilitation, which means recovering a system that has not returned to its original state or is as healthy as it would have been if it had been fully restored [5].

The Magdalena-Eslava river system is one of the last non-piped rivers remaining in the MCMA. Given its contribution to the water supply in its upper reaches and the potential to recover some of the environmental services provided by the system, there is significant interest in the possibility of improving the water quality in the lower parts of its course. The aim of this study was to evaluate the Magdalena-Eslava river system in terms of the amount and quality of water, based on physicochemical and microbiological attributes that it offers the community. Accordingly, the study was performed based on historical and current data. As one of the last urban rivers, this study provides an opportunity to pose alternatives for more efficient water use.

### Study area description

The study area, which is located in Mexico City, comprises the Magdalena and Eslava rivers. The river system covers two climatic regions. The lower area, which reaches to 3,050 masl, presents a temperate sub-humid climate with a summer rainy season and average temperatures that oscillate between 12 and 18°C. The upper basin has a semi-cold climate and a rainy season during the summer in areas higher than 3,050 masl, with an annual mean temperature between 5 and 12°C [6]. The rainy season ranges from May to October, with a monthly precipitation in excess of 250 mm in July [7]. The annual precipitation in most of the area is between 1,200 and 1,500 mm [8].

The Magdalena River originates in *Sierra de Las Cruces* from springs and runoff from the *La Palma*, *El Gavilán* and *El Muñeco* mountains in a location called *Cieneguillas*. The Magdalena River Basin is actually a sub-basin of the Basin of Mexico (hereafter called the Magdalena River Basin) and belongs to what is legally known as the *Zona Protectora Forestal Cañada de Contreras*, which was declared in 1932. The protected zone includes a fringe of 12 km from the river's origin, with a 500 m buffer on each side of the river, as declared in 1947 [9], [10].

The Magdalena river basin extends from 2,570 to 3,870 masl [11], covering an area of 29.80 km^2^. The Eslava River, which is the basin's largest tributary, flows through an area of 24.05 km^2^, with an altitude descending to 2,510 masl at the urban limit.

**Figure 1:**
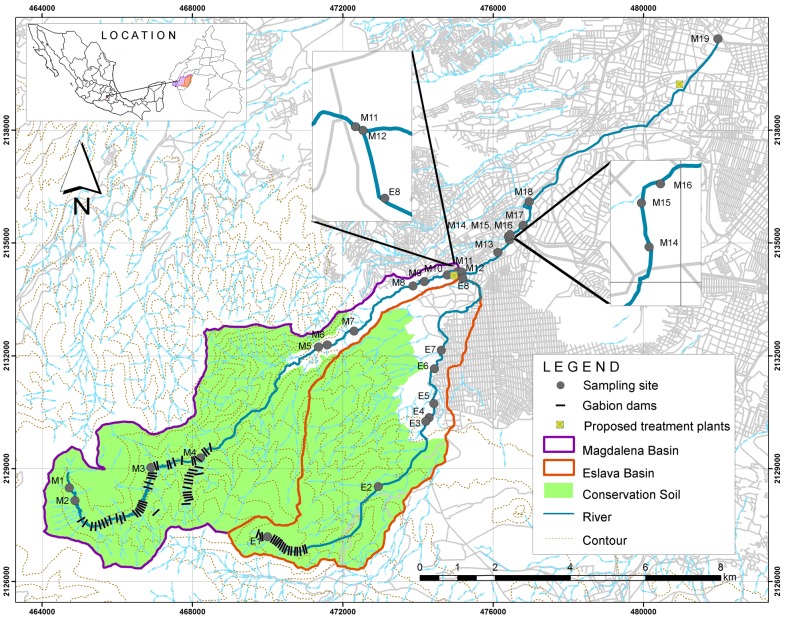
Study area, including the Magdalena and Eslava rivers within the Basin of Mexico and the Mexico City Metropolitan Area.

The Magdalena River is 28.2 km long and flows 14.8 km through a natural forested area known as the Conservation Soil, with an additional 13.4 km crossing through the urban area. In the urban section, 8.8 km of river flows as an open canal, and 4.6 km is piped [12]. The Eslava River is 13.7 km long, with 10.96 km in the Conservation Soil and 2.74 km in urban areas [12]. The basins of the Magdalena and Eslava rivers are shown in Figure 1.

The Magdalena Basin, known as *Cañada de Contreras* in *Sierra de las Cruces*, is the only drainage of significant magnitude [13] within the Basin of Mexico and is primarily classified as temperate sub-humid. Despite its location in a peri-urban area, the Magdalena Basin serves as an important refuge for biodiversity and is home to 1,175 species, of which 212 are considered useful and 39 are listed in the at-risk category [14].

Three vegetation communities are present in the area, according to Ávila-Akerberg (2009) [8] and Nava (2003) [15]. The dominant vegetation types are *Abies religiosa* forest (46%) from 2,750 to 3,500 masl, *Pinus hartwegii* forest (29%) from 3,420 to 3,800 masl, *Quercus* (8.3%) and mixed forest (1.3%) from 2,600 to 3,370 masl, grassland (7.2%), and cloud forest (0.2%). Each type of forest produces different rates of runoff, as calculated by Jujnovsky et al. (2010) [16]. The *Abies* area produces 10,944,800 m^3^ of water per year, the *Pinus* forest produces 6,878,000 m^3^ of water per year, and the mixed oak forest generates 3,217,500 m^3^ of water per year.

The natural vegetation cover has been altered as a result of human activities, resulting in an increase in erosion and the reduction of water and soil humidity retention. Therefore, water flows rapidly during flood events in the lower parts of the basin [13]. The forested area southwest of Mexico City represents the most important surface water source and groundwater recharge area. This area supplies water and regulates both flood events and the microclimate in the MCMA. Urban expansion represents a threat to the Conservation Soil area, which currently provides ecosystem services, such as fresh water.

At the request of the City, the *Gobierno del Distrito Federal* through its Environmental Ministry (*Secretaría del Medio Ambiente*), the two main universities located in Mexico City, the National Autonomous University of Mexico (UNAM) and the Metropolitan Autonomous University (UAM), generated a Master Plan to Rescue the Magdalena and Eslava Rivers [12]. This plan was developed by an interdisciplinary team that included engineers, architects, ecologists, biologists, architects, geographers, and sociologists, who proposed to rehabilitate the river as an urban area that could be used as a community recreation area while recovering the water quality and the watercourse running through the city [12]. A second project developed a system of indicators to measure the improvements resulting from the actions taken as a result of the Master Plan [17], which was not completely implemented.

The topic of river ecosystem recovery and rehabilitation has grown in relevance, receiving increasing attention in the last two decades. From an urban perspective, rivers are crucial for water supply, flood and drought regulation, storm-water management, water quality improvement, and erosion control; additionally, rivers serve as urban wildlife refuges. Overall, rivers can serve a range of purposes from supplying water to offering locations for social and recreational activities; hence, rivers often account for a significant fraction of the few recreational green areas in large cities [18], [19].

## Methods

To determine the water flow, historical data were collected and analyzed (1973–2010); this approached allowed us to discern important variations due to seasonal or management effect in the river system. Based on current water quality measurements (2008–2012), we describe the actual condition of the river based on physicochemical and microbiological variables. The methods described in this section were applied.

### Water flow

A flow analysis was conducted based on historical data recorded at the Santa Teresa hydrometric station (26440) to understand the water quantity and variability of flow at the only existing station of the National Water Commission. This hydrometric station is located after the junction of the Magdalena and Eslava rivers. As mentioned above, the Eslava is the main tributary of the Magdalena River, which also receives inflows from additional small creeks and natural run-off, combined with the discharge of wastewater from the surrounding urban area.

Daily information was obtained from the official flow surveys recorded at the Santa Teresa hydrometric station (26440) and was published in the National Data Bank of Surface Water [20] of the National Water Commission (*Comisión Nacional del Agua*). The data were analyzed for consistency and to detect and correct deviations from the historic series based on the double mass curve method [21], [22]. The series of daily data spanning 38 years were then plotted, and two periods for the flow regime were identified.

We calculated the average monthly flow for each regime, i.e., the natural and the regulated areas, so we could compare the regulating effect of water infrastructure on the river. Finally, we plotted the average, minimum and maximum annual flows to observe the river's evolution over time.

### Experimental design

After a general survey of the area, sampling sites for water quality evaluation were selected at places with little human or animal influence in the natural area and at others with inputs of urban runoff and wastewater in the urban area. Based on an initial diagnosis performed in 2008 and 2009 [12], [17], [23], the sampling locations were evaluated for redundancy in terms of the information provided. We computed a correlation matrix for the 19 sampling locations. After 2009, the sampling protocols included ten sampling sites. If two locations had a Pearson's correlation above 0.6 and were neighbors of each other, one was eliminated. This procedure allowed us to reduce the number of sampling locations from 19 to eight stations. We then added two sampling locations a few meters upriver of the intake of the water treatment plants that are located along the river. These ten stations were sampled from 2010 to 2012. The sampling covered the dry and rainy seasons, as shown in Table 1. The selected sampling sites, identified as representative of the Magdalena and Eslava rivers, are depicted in Figure 1. Fieldwork permission was given by the *Secretaría del Medio Ambiente* (SMA) and by the *Comisión de Recursos Naturales* (CORENA), the local authority that belongs to the *Gobierno del Distrito Federal* (GDF).

**Table 1:** The sampling design for the Magdalena-Eslava river system.

Variable	Dry season	Rainy season
Magdalena River		
Physicochemical	2008, 2009, 2010, 2012	2008, 2009, 2010, 2011, 2012
Indicator bacteria	2008, 2009, 2010, 2012	2008, 2009, 2010, 2011, 2012
Pathogens	2008, 2009	2008, 2009, 2010
Coliphages	2008, 2009	2008, 2009
Virus	2008, 2009	2008, 2009
Protozoa	2008, 2009	2008, 2009
Eslava River		
Physicochemical	2011, 2012	2010, 2011, 2012
Indicator bacteria	2011, 2012	2010, 2011, 2012
Pathogens		2010

The behaviors of the two rivers were plotted independently, where the Magdalena River (main course) is presented with a five-year data set (2008–2012), while its tributary, the Eslava River, is shown with a two-year data set (rainy season 2010, 2011–2012) in Table 1.

### Physicochemical analyses

As part of a long-term water quality monitoring program in the Magdalena Eslava river system, the attributes of temperature (°C), pH, electric conductivity (µS/cm), turbidity (NTU), total dissolved solids (TDS, mg/L), dissolved oxygen (DO, mg/L), DO saturation percentage, total phosphorus (total P, mg/L), orthophosphate (P-PO_4_, mg/L), total nitrogen (total N, mg/L), ammonia (N-NH_3_, mg/L), and nitrates (N-NO_3_, mg/L) were measured.

The physicochemical variables of temperature, pH, electric conductivity, turbidity, total dissolved solids and dissolved oxygen were measured *in situ* over the course of five years. All measurements were made using a Multiparameter Water Quality Sonde YSI 6600-M (Yellow Springs, OH, USA). Five-hundred-milliliter water samples were taken and analyzed in triplicate for nutrients using a spectrophotometer HACH Model DR2400 (Loveland, CO, USA) following the HACH manual [24]. Total P was measured following the method of PhosVer3 by acid digestion USEPA (0.06 to 3.50 mg/L P) and the molybdovanadate method (1 to 100 mg/L PO_4_^3−^). Orthophosphate was measured using the amino acid method (0.23 to 30.00 mg/L PO_4_^3−^), and ascorbic acid was measured using PhosVer3 USEPA (0.02 to 2.50 mg/L PO_4_^3−^). Total N was measured using the persulfate digestion method (10 to 50 mg/L N) and (0.5 to 25.0 mg/L N). Ammonia was measured following the silicate method (0.02 to 2.50 mg/L NH_3_-N), and nitrates were measured using the cadmium reduction method USEPA (0.01 to 0.50 mg/L NO_3_-N) [24].

### Microbiological analyses

Samples for microbiological analyses were taken at the same time and location as those collected for the physicochemical analyses.

#### Bacteria counts

One-liter samples for bacteriological analyses were collected from selected sites using polypropylene sterile flasks. Samples taken in triplicate were transported and stored at 4°C according to the American Public Health Association standard procedures [25], [26]. Microbiological samples were processed within 24 h of collection. All samples were analyzed in triplicate, and the results were reported in colony-forming units (CFUs) [27], [28].

Two bacteriological variables, including fecal coliform (FC, UFC/100 mL) and fecal enterococci (FE, UFC/100 mL), were measured constantly during the period from 2008–2012. Total coliforms (TC, UFC/100 mL) were determined only for the period 2008–2009. Positive samples were processed to identify the pathogenic and opportunistic bacteria.

Water samples were analyzed following the standard membrane filtration procedures to enumerate three bacterial types, namely, FC, FE, and TC. Membrane filters (0.45 µm cellulose acetate, Millipore MF type HA) were placed on M-FC agar for FC, KF Streptococcus agar for streptococci/enterococci, and m-Endo agar MF for total coliforms. Incubation was performed with a WTB Binder brand incubator at 35°C for 24 h for enterococci and total coliforms and at 44.5°C for 24 h for fecal coliforms, according to APHA [25], [26]. The results are reported as colony forming units (CFUs).

#### Bacteria identification

Gram-stain and biochemical tests were performed to identify bacteria using a semi-automatic DADE MicroScan, AutoSCAN-4 (DADE International. West Sacramento, CA) [26]. Positive samples from water filtration isolates were selected based on morphology. Five colonies of each specific morphology were selected and identified by the MicroScan Auto SCAN-4 DADE (West Sacramento, CA).

Additional microbiological variables were analyzed during the period 2008–2009 and during the rainy season of 2010. We evaluated these variables because of their importance and relation to gastrointestinal diseases as a result of their prevalence in water in Mexico. These microorganisms included coliphage, enterovirus (EV), adenovirus F human serotypes 40 and 41 (AdV F), *Cryptosporidium parvum* oocysts and *Giardia lamblia* cysts.

#### Coliphage

Coliphages were quantified in duplicate following the double-layer agar method based on the International Standard Water quality-Detection and enumeration of bacteriophages (ISO 10705-1, 1995) [29]. The plaques were quantified after the incubation period, and the results were reported in plaque forming units per milliliter (PFU/mL) [30].

The method detection limit was determined following the double-layer agar technique. Serial dilutions were performed using an MS2 phage with a known titer of 10^8^ PFU/mL and *E. coli* K12– Hfr 3000 (ATCC 23631) as the host bacteria. The detection limit for this method was determined to be between 10 and 1 PFU [31].

The coliphage quantification with the *E. coli* Hs (pFamp) as host bacteria was performed in duplicate using the adaptation USEPA “Method 1601: Male-specific (F+) and Somatic Coliphage in Water by Two-step Enrichment Procedure)” [29], [30].

#### Virus determination

For the detection of viruses and protozoa, 10-L water samples collected from the natural sites were concentrated by ultrafiltration following the method of Polaczyk et al. (2008) [32].

Enterovirus (EV) quantification was performed in duplicate on the water samples using RNA extraction techniques based on the RNA viral QIAamp (QIAGEN) mini kit. To quantify the genome number of EV, the basic protocol reported by Monpoeho et al. (2000) [33] was followed.

To obtain the AdV F genome number, the protocol by Xagoraraki et al. (2007) [34] was followed.

#### Protozoa quantification

*Cryptosporidium parvum* and *Giardia lamblia* were detected and quantified by immunofluorescence microscopy.

One milliliter of each sample (direct for wastewater or concentrated for clean samples) was analyzed in single samples using an indirect immunofluorescence technique in liquid phase according to Method 1623 [35], as modified by Rangel-Martínez [36] and Tapia-Palacios [37]. To count the (oo)cysts in the samples, an Axiostar Plus (Carl Zeiss, Gottingen, Germany) fluorescence microscope was used. Total scans of each slide were performed at 20X and 40X and, in special cases, at 100X for confirmation.

### Statistical Analyses

The data gathered during the study were analyzed in a spatial and temporal context. Because the sampling locations were located along river courses, we assumed that the spatial coordinates were one-dimensional. In this coordinate system, we identified each site by a site code and by the distance from the first sampling location in the uppermost part of each river. We fitted generalized linear models (GLMs) with the adequate link function for each attribute [38]. For example, for each variable whose histogram clearly skewed to the left, we fitted GLMs with Gamma error and a logarithmic link function. These analyses were performed to explore the association between the physicochemical and biological variables, using site, season and year as covariates. Such analysis allowed us to detect significant effects of the covariates in each of the attributes considered in the analysis. We also created interaction plots to graphically depict the spatial behavior of the values of the variables along the downhill path of the Magdalena and Eslava rivers and at their confluence. Separating the courses in this way allowed us to compare the Magdalena and Eslava rivers before their merging point and to detect whether differences were present in the water quality variables relative to downriver points.

## Results and Discussion

### Water flow

Despite their relatively small size, the Magdalena River and its main tributary, the Eslava River, provide water to part of the southern area of Mexico City and are the main surface water source for the city, representing more than half of the surface water used. Several studies have been conducted to estimate the amount of water provided by the Magdalena River. In the 1950s, when water sources for the city were investigated, the Magdalena River registers showed a mean flow of 0.33 m^3^/s or 10,400,000 m^3^/yr [39]. The mean water volume for the Magdalena River was reported in 1975 as 6,970,000 m^3^, equivalent to 0.22 m^3^/s, and that for the Eslava River was 3,578,000 m^3^, equivalent to 0.11 m^3^/s [40]. These volumes total 0.33 m^3^/s, with a total volume for potential use of 10,548,000 m^3^/yr.

A first view of the hydrologic ecosystem services in terms of water quantity and quality with runoff estimates per environmental unit gives an annual production for 2002–2003 of 21,538,250 m^3^, equivalent to 0.67 m^3^/s, which is similar to the 0.58 m^3^/s reported at the hydrometric station in 1999. From 1999–2001 [41], the average flow was 0.70 m^3^/s, of which 81% corresponded to baseflow and 19% to surface runoff, providing water for approximately 118,237 inhabitants. In a complementary study, Jujnovsky et al. (2012) [42] assessed the water supply from the Magdalena River Watershed, determining the water balance based on the SWAT model, and obtained a water provision of 18,400,000 m^3^/yr. The baseflow contribution accounted for 85%, while the surface runoff was approximately 15%, providing drinking water for approximately 78,500 inhabitants and being capable of supplying 153,203 potential beneficiaries.

#### Natural and regulated flow regimes

**Figure 2:**
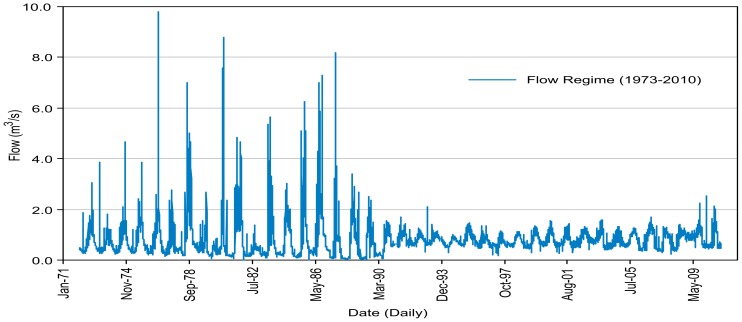
Daily volumetric flow regime (1973–2010) measured at the hydrometric station Santa Teresa (26440).

Our study shows the mean annual flow in the hydrometric station to be 0.78 m^3^/s, with contributions of 0.45 m^3^/s (57%) from the Magdalena River and 0.34 m^3^/s (43%) from the Eslava River. The daily regimen is shown in Figure 2, based on historical data from 1973 to 2010, clearly depicting two periods. The first period is from 1973 to 1989, which represents the natural flow regime (NFR), and the second period is from 1990 to 2010, which represents a regulated flow regimen (RFR). This graph shows a decreasing flow and less seasonal variability in the maximum and minimum under the RFR.

The natural flow regime achieved rates of 0.90 m^3^/s per year, surpassing the 2 m^3^/s during 16 of the 17 years identified as NFR. Starting in 1990, the maximum flow rarely reached 2 m^3^/s. The minimum regulated flow of the second period is three times higher than the natural minimum flow, varying from 0.13 to 0.37 m^3^/s, that is, the minimum flow increased by 176%. In contrast, the maxima were reduced by 69% (Figure 2). Regulation structures, such as gabion dams, increase the minimum flow due to retention and water storage. At present, more than 60 regulation structures have been registered [12], as shown in Figure 1. These structures were constructed for different objectives, such as decreasing the flow velocity, creating micro wetlands and diminishing sediment volumes, among others [12]. These hydraulic structures are not recommended because they function as frontiers that break the biological continuity of lotic systems [17].

**Figure 3:**
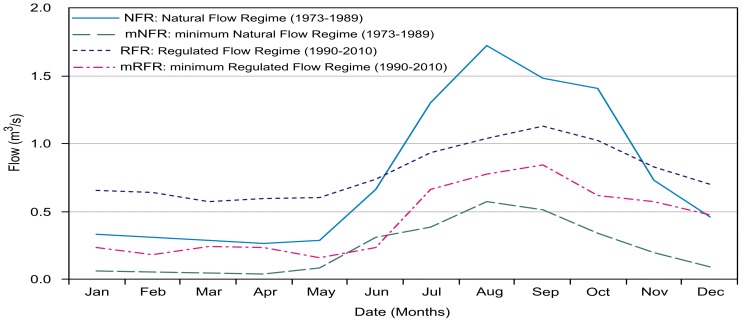
Interannual variation of the NFRs, RFRs, mNFRs and mRFRs measured at hydrometric station 26440.

The hydrometric information is depicted in the curves of average monthly flow (Figure 3). The NFR includes the dry season (January to May), with a mean measurement of 0.29 m^3^/s and a lower flow in April of 0.26 m^3^/s. The rainy season shows an increase in flow from June to August and a decline in December (0.66, 1.72 and 0.46 m^3^/s, respectively). At the end of the 1980s, the basins of the Magdalena and Eslava rivers experienced flow variations, as observed in Figure 3. In the dry season, the RFR curve increased 128% compared with the natural regime (NFR). The RFR value for April was 0.60 m^3^/s, compared with 0.26 m^3^/s for the NFR in the same month. In the rainy season, the RFR curve diminished with respect to the NFR by 30% from July to October.

Figure 3 shows the changes experienced by the river before and after the hydraulic regulation in the periods mentioned previously, which is defined for four average monthly flow regimes. The inertial behavior of the minimum natural flow regime (mNFR), shows that the dry period (January-May) minimum flow was 0.06 m^3^/s, except in March-April, when the minimum flow was 0.04 m^3^/s. Starting in the rainy period, the minimum flow responded to the natural increase from June-August (a maximum flow of 0.57 m^3^/s) until declining over the period of September to December from 0.51 to 0.09 m^3^/s.

The minimum regulated flow regime (mRFR) differs from the natural condition (mNFR curve), with a greater effect observed during the dry season. In March and April, the minimum natural flows increased to 490% (from 0.04 to 0.24 m^3^/s) and then increased 202% in January, February and May. From July to October, the mRFR curve increased 64% from its original condition (1973 to 1989); in November and December, the flows increased from 0.20 and 0.09 to 0.57 and 0.47 m^3^/s, respectively. This increase can be attributed to the hydraulic structures and to external factors, such as wastewater discharge from irregular settlements in the lower drainage basins and surface runoff during the rainy season. The hydrological histogram (Figure 3) shows the discrepancy in hydrological conditions: under low-flow conditions, the mRFR curve corresponds to the NFR conditions. Therefore, the minimum regulated behavior mimics a natural condition.

**Figure 4:**
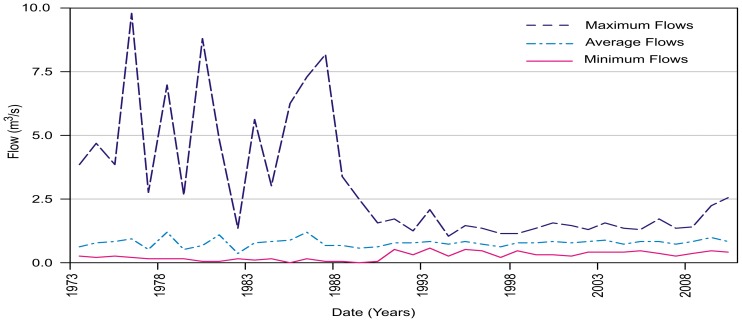
Annual volumetric flow regime at the outlet of the Magdalena and Eslava river basins. Average, maximum and minimum flows.

Figure 4 depicts the annual flow over a 38-year period. In the first period (1973–1989), the maximum flow reached 10 m^3^/s, with a mean annual maximum of 4.94 m^3^/s. In the 1990s, the development of hydraulic infrastructure in the river caused a laminar flow effect. During the second period, from 1990 to 2010, the maximum flow decreased 69% (relative to 2.53 m^3^/s as the maximum), with a mean annual maximum flow of 1.52 m^3^/s.

The behavior of the mean annual flows (Figure 4) also reflects the effect of regulation. Although the mean flow in both periods is very similar, namely, 0.77 and 0.79 m^3^/s, its volume has not changed significantly. Over time, extraction from the river has been balanced by wastewater inputs into the river, which has led to an increase of 0.02 m^3^/s. The exchange of clean water for untreated wastewater has significantly degraded the environmental condition of the river.

There are only two direct discharge points into the river over the course of the distance from its origin to the treatment plant; in contrast, after the treatment plant, 58 inputs are known [12]. Irregular settlements are problematic because they often lack a sewer system, thereby allowing wastewater to flow directly into the rock or soil and hence toward the lower discharge area where the river is located.

**Figure 5:**
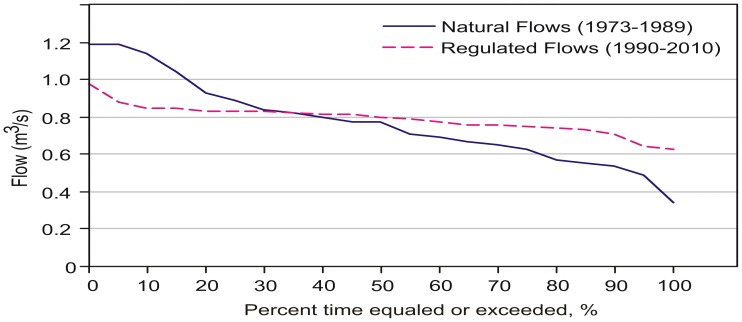
Duration curve of annual natural and regulated flows, Magdalena river basin, 1973–2010.

In Figure 5, a historical annual comparative analysis illustrates the natural flow from 1973 to 1989 in two flow-duration curves; during this time, the annual mean flow at the lower point of the Magdalena river was at least 0.54 m^3^/s more than 90% of the time, which represents the probable flow that the river used to supply along the year. High flows with flows of approximately 1.14 m^3^/s are present during a very short period (10% of the times). In the 1990s, the development of hydraulic infrastructure in the river caused a laminar flow effect; therefore, in the curve for the regulated period from 1990–2010, the mean flow at the river is at least 0.71 m^3^/s for 90% of the time, which is an increase of 31% in comparison with the previous natural minimum; consequently, a 25% decrease in high flows is observed. It is also possible to observe changes to the slope of the natural flow curve (reference) with respect to the regulated flow curve, specifically, changing from 0.73 to 0.24%.

The behavior of the natural curve of collected frequencies (1973 to 1989) depicted in Figure 5 is representative of mountain rivers; therefore, based on the flow duration curve observed for the regulated period (1990 to 2010), a new behavior is observed. That is, the river is behaving if it were a floodplain river, where changes are less apparent. Generally, this response is due to changes to the flow of the Magdalena River as a result of anthropogenic activities, with an associated increase in the concentration of several water quality variables.

**Figure 6:**
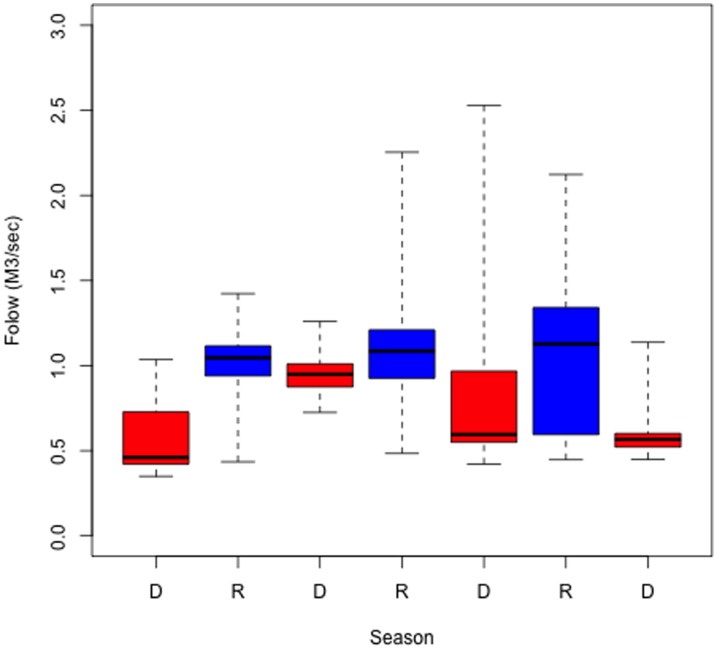
Water flux distribution from 2008–2010 grouped by season.

Figure 6 shows the distribution of water flux for the rainy and dry seasons for the years 2008–2010, starting with the dry season from January-April 2008, followed by the rainy season of 2008, the dry season from November 2008–April 2009, and so on. The figure demonstrates that the mean value is relatively constant for dry and rainy seasons, while the variability changed during those same years; hence, no pattern can be observed for the changes in variability. This is related to the high variability of the amount of rain falling in the upper parts of the basin, particularly during the rainy season of 2010. The low variability for the dry season from November–December 2010 is due in part to the small amount of data for this time lapse.

### Water quality

#### Physicochemical and microbiological analyses

**Figure 7:**
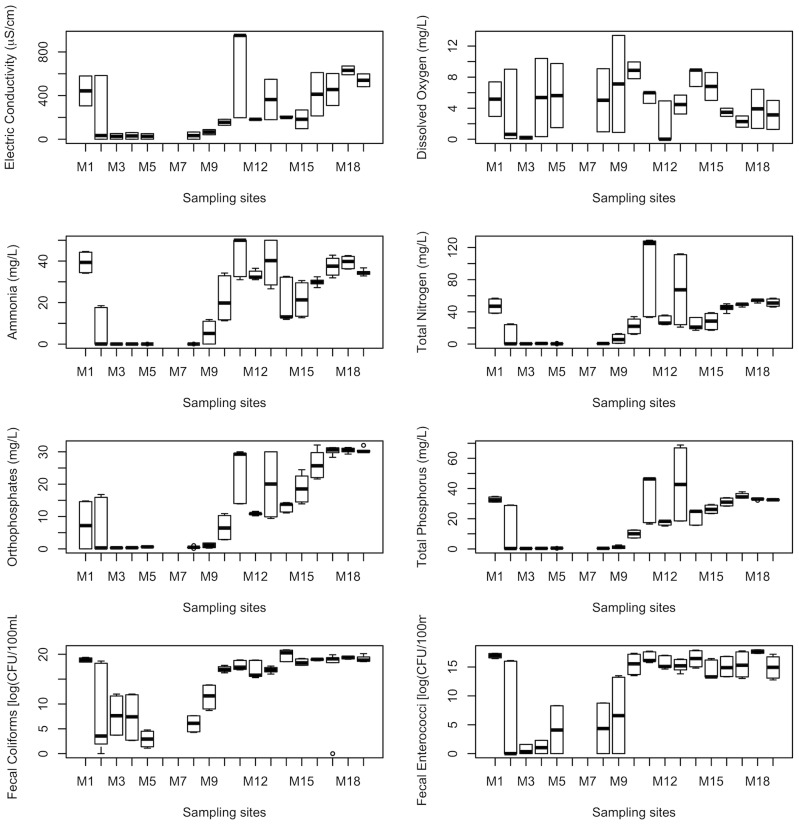
Spatial trend for the eight main water quality variables in the Magdalena River, based on 19 sampling stations.

**Figure 8:**
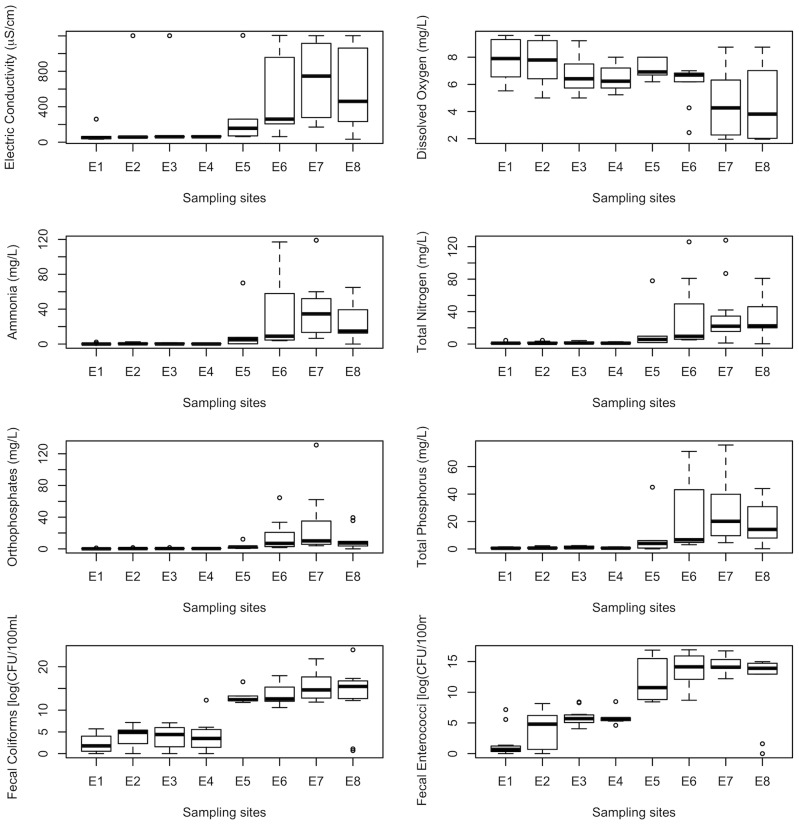
Spatial trend for the eight main water quality variables in the Eslava River, based on eight sampling stations.

The spatial and temporal analyses for the physicochemical variables and nutrients are presented in Figures 7 and 8. These variables are those most representative of river water quality, such as electrical conductivity, dissolved oxygen, ammonia, total N, orthophosphates and total P, as well as fecal coliforms and fecal enterococci. These variables are considered representative because they have the highest absolute value in the factor loadings of the first three principal components. The factor loadings for these attributes account for more than 80% of the total variability of the Magdalena River system [43].

Although the number of samples at each site was not the same, it only affects the variance of the mean values of the measured water properties. This effect has been considered and corrected in all of our analyses by dividing the mean value estimates by σ/√n.

The GLM models fitted to the water quality variables [38] indicate statistically significant differences for all of the variables, except dissolved oxygen, in the upper and lower sections of both rivers. The point of change was the location at which human activities and settlements began on either or both river banks. Water quality variables sampled upstream of these urban areas fell within acceptable limits for use as a water supply. The values gradually fell to lower quality as the sampling sites became increasingly affected by the wastewater generated in the irregularly inhabited areas. The spatial trends along the river gradient for the eight most-relevant water quality attributes are shown as box plots in Figure 7 for the Magdalena River and in Figure 8 for the Eslava River.

The plot shows a change point at site M9, which is located at the border between the natural and urban areas: here, increasing concentrations of all values can be observed, except that of dissolved oxygen, which decreases. The highest concentrations were detected at site M15, where part of the water is piped to the city's drainage system. These values then decrease slightly at sites M17 and M18, where other effluents with residual water from the southern area of the city join the waterway.

In the Eslava River, all variables except dissolved oxygen are significantly different between the natural and urban areas, a change that occurs somewhere between E5 and E6. Further, all variables exhibit stable values until the confluence point. The Eslava River has lower water quality than the Magdalena River does at the confluence point, particularly in terms of electrical conductivity, the value of which is six times higher in the Eslava River; additionally, the value of total N is three times higher, while that of total P is twice the concentration of that in the Eslava River. The fecal enterococci values were three times as large on a log scale, which means that this measurement is 1,000 times greater. Please note this difference in the values of the axes on the graphs.

According to Mexican standards, several attributes surpass the NOM-001-SEMARNAT-1996 [44] limits for water discharged to Mexican water bodies. The total N exceeds these levels from M15 to M18 and is double the limit at E8 in the Eslava River. At E7, the values are slightly above the limit.

Because both rivers are used as surface water sources for human use and consumption, the LFDMA [45] limit is 1,000 FC per 100 mL. Taking this into account, the fecal coliforms surpass the limit and require treatment before distribution through the southern parts of the city. The standards are met at the Potabilization Plant La Magdalena, but there are many water intakes near settled populations in an irregular land ownership situation, which means that these populations are exposed to high levels of bacteria; this is an important risk factor that should be taken into further account.

#### Bacteria identification

The 164 bacterial isolates and identifications obtained from 516 water samples taken from the Magdalena River in the period 2008 to 2010 are presented in Table 2. These belong to nine different families and 18 genera [26], [46]. The most abundant genera were *Enterococcus*, *Escherichia coli*, *Enterobacter* and *Klebsiella*. From the Eslava River, 27 bacterial isolates and identifications were obtained from 120 water samples taken during 2012. These belong to four different families and seven genera, as reported in Table 3.

**Table 2:** The bacteria identified in the Magdalena River water samples in 2008, 2009 and 2010.

	Sites																			
Isolated microorganisms	M1	M2	M3	M4	M5	M6	M7	M8	M9	M10	M11	M12	M13	M14	M15	M16	M17	M18	M19	
n	24	24	6	24	42	42	18	18	42	42	6	6	42	24	42	24	42	24	24	Total
Moraxellaceae																				
* Acinetobacter* spp.																		1		1
Aeromonadaceae																				
* Aeromonas* sp.				1										1		1	1	1		5
Burkholderiaceae																				
* Burkholderia* sp.								1												1
Alcaligenaceae																				
* Bordetella* sp.								2												2
Enterobacteriaceae																				
* Citrobacter* spp.													1				1			2
* Enterobacter* spp.	1	1			1			3	1		2	1	3	2		1		1	2	19
* Escherichia* sp.	2	1			2			1	2	1	2	3	2	1	1	3	3	3		27
* Hafnia* sp.	1																			1
* Klebsiella* spp.								1	1		1	2	1		1	2	2	2		13
* Proteus* sp.					1															1
* Providencia* sp.			1	1																2
* Salmonella* sp.								1												1
* Serratia* spp.									2											2
* Yersinia* sp.						1		1												2
Leuconostocaceae																				
* Leuconostoc* sp.					1															1
Pseudomonadaceae																				
* Pseudomonas* sp.			2	3	1				1		1				1					9
Vibrionaceae																				
* Vibrio* sp.									1			2	1	2	1	1				8
Enterococcaceae																				
* Enterococcus* spp.	4	2		5	5	1	1	6	5	2	6	3	2	6	2	6	6	5		67
Total	8	4	3	10	11	2	1	16	13	3	12	11	10	12	6	14	13	13	2	164

**Table 3:** The bacteria identified in the Eslava River water samples in 2010.

	Sites								
Isolated microorganisms	E1	E2	E3	E4	E5	E6	E7	E8	Total
n	15	15	15	15	15	15	15	15	
Burkholderiaceae									
* Ralstonia* spp.				1					1
Bacillaceae									
* Bacillus* spp.	1								1
Enterobacteriaceae									
* Escherichia* spp.		1	1	1	1	1	1	2	8
* Enterobacter* spp.				1					1
* Klebsiella* spp.						1	1	2	4
* Yersinia* spp.								1	1
Enterococcaceae									
* Enterococcus* spp.	1	1	1	1	1	1	1	4	11
Total	2	2	2	4	2	3	3	9	27

Therefore, a variety of bacteria belonging to a total of 20 different genera were identified in the river system crossing the southern area of Mexico City. The most prevalent bacteria were from the Enterococcaceae family, *Enterococcus*, and from the Enterobacteriaceae family, *Escherichia*, *Enterobacter* and *Klebsiella*. Although these results serve as an initial approach to the identification of bacteria, it is important to note that all of these bacteria are common to the human gut and can be opportunistic pathogens that may cause illness to immune-compromised people who access this water when untreated.

#### Coliphage, virus and parasite analyses

The presence of coliphage was measured by bacterial hosts in samples from the urban zone; this finding is consistent with the inputs of raw and untreated wastewater to the Magdalena River (Table 4). As host bacteria, *E. coli* K12-Hfr registered the higher sensitivity, although larger counts were obtained for *E. coli* Hs (pFamp). It is important to note that there are two infection mechanisms of interest, either by adherence to the cell wall or infection via pilli, as in the coliphage F-specific [47], [48], [49], [50]. Therefore, the host is more susceptible to infection and shows higher counts.

**Table 4:** The geometric mean, standard deviation in log scale, maximum and minimum of Coliphage, Enterovirus, Adenovirus, *Cryptosporidium parvum* oocysts and *Giardia lamblia* cysts in the Magdalena River water from 2008 to 2009.

		Sites							
		M1	M5	M8	M11	M12	M17	M18	M19
Coliphage with host	Geometric mean	<1	<1		39	52	57	22	114
K12	Standard deviation	<1	<1		17	18	58	51	14
PFU/mL	Min-Max	<1	<1		29–65	27–65	9–140	7–110	97–127
	n	4	4		4	4	6	6	6
Coliphage with host	Geometric mean	<1	<1	<1	4	7	3	3	33
Hs	Standard deviation	<1	<1	<1	10	7	10	3	26
PFU/mL	Min-Max	<1	<1	<1	<1–18	<1–17	<1–20	<1–7	18–80
	n	4	4	2	4	4	6	6	6
Enterovirus Gen/mL	Geometric mean	<1	<1	<1	<1	<1	<1	<1	<1
	Standard deviation	<1	<1	<1	<1	<1	<1	<1	<1
	Min-Max	<1	<1	<1	<1	<1	<1	<1	<1
	n	3	5	6	4	4	6	4	4
Adenovirus Gen/mL	Geometric mean	10	<1	<1	12974	7299	98294	7305	97084
	Standard deviation	577	<1	<1	46297	28951	253772	10157	35432
	Min-Max	<1–1000	<1	<1	1130–930464	982–63008	24930–557800	1618–25186	71486–144840
	n	3	5	4		4	6	4	4
Oocysts	Geometric mean	<1	32	<1	6784	21	575	4244	<1
*Cryptosporidium*	Standard deviation	<1	707	<1	14849	5196	9504	2121	<1
*parvum*/L*	Min-Max	<1	0–1000	<1	2000–230002	<1–9000	<1–19000	3000–6000	<1
	n	2	2	1		3	3	2	2
Cysts *Giardia*	Geometric Mean	1000	71	<1	18	18	30	89	<1
lamblia/L*	Standard deviation	<1	3536	<1	2828	3464	16166	5657	<1
	Min-Max	1000–1000	<1–5000	<1	<1–4000	<1–6000	<1–28000	<1–8000	<1
	n	2	2	1	2	2	3	2	2

*Cryptosporidium parvum and Giardia lamblia* detection limit 1 (oo)cyst/mL [37]; Adenovirus (AdV) detection limit 10 genome copies [34]; Enterovirus (EV) detection limit 10 genome copies [74]; FRNA specific bacteriophage detection limit 1 PFU/mL [30], somatic bacteriophage detection limit 1 PFU/mL [30], [34].

In the natural area sites, no coliphage presence was detected, which suggests that perturbation does not have a detectable impact using this method. Generally, the presence of a phage indicates fecal contamination by a virus; however, this level of analysis is not conclusive because it cannot establish the contaminants' origin, namely, whether human or animal. In urban areas, coliphages are present from the transition zone between the natural and urban zones, where the highest density was detected at site M19; hence, the number of phages increases with human perturbation and influence.

Both adenovirus (AdV F) and enterovirus (EV) exclusively affect humans; their presence indicates human fecal contamination [51], [52]. Once these viral particles have been excreted into the environment, their number cannot increase because the presence of the cell host is required for their replication [53]. Therefore, the detection of AdV and EV reflects the fact that in the human population, the virus is circulating and replicating. This constitutes a threat that must be considered regarding the river because these viruses can cause infections to susceptible individuals, yielding viral gastroenteritis.

AdV F replicates quickly in the gastrointestinal track, so the amount of excreted viral particles is on average 1×10^10^/g in feces [53]. Such infections mainly occur in children under five years of age because adults who were infected as young children often acquire immunity [52]. Nevertheless, if adults are exposed to viral particles, they can be asymptomatically reinfected, with a lower amount of viral particles excreted in their feces. This can favor the dispersion of AdV F because these individuals will not be cautious in their behaviors because they are asymptomatic.

People can present infections and acute gastroenteritis as a result of AdV F [54]. It has been estimated that this virus is the cause of 9 to 12% of acute gastrointestinal cases in children under five years of age in Mexico; however, based on the lack of follow up regarding this type of pathogen and the lack of updated epidemiological information (few studies have been conducted since 1985), this may be an underestimation [55]. Due to the relevance of this virus as a gastroenteritis-causing agent, it is considered the second-most important virus after rotavirus worldwide [56]; therefore, it is important to monitor the prevalence of this virus both in the human population and in residual water as an indicator of what is infecting a given population.

According to our results (Table 4), AdV F is present at both the natural and urban sites, indicating human fecal contamination in both locations. The presence of AdV F at the river origin (M1) could represent an isolated contamination event because it was only detected at a low density in the samples from this site. The presence of human fecal contamination coincides with the irregular settlements that discharge their wastewater directly to the river. Because the system once flowed continuously, the viral contamination issue is likely a recent phenomenon. Wastewater discharge is constant; unsurprisingly, the higher amounts of AdV F viral particles were detected in the urban zone due to wastewater discharge without proper treatment [54], [57].

It is important to note that the number of AdV F particles can represent an important mechanism of exposure to the population, primarily for children under five, who may be more likely to come in direct contact with the water in urban areas; accordingly, this exposure can lead to moderate to severe gastroenteritis [53]. For the exposure dose, a point estimate was reported by Crabtree *et al.* [58], who considered AdV 4 as a surrogate because no AdV 40/41 dose-response has been published. Based on this finding, a 2 infectious viral particle can be considered the probability for infection based on an average dose of 1.66 [59].

Considering that the detection limit of the protocol applied for AdV 40/41 quantification was ten genome copies, as determined by serial dilutions from the positive control, it would not be possible to identify a hazard related to AdV 40/41. Nevertheless, the fact that the genome detection rate is commonly larger than the number of viral particles [60] clearly demonstrates that the detection capacity of 10 genome copies is adequate for the identification of adenovirus as a hazard.

*Cryptosporidium parvum* and *Giardia lamblia* are the most common intestinal protozoa in the environment, commonly infecting both humans and domestic and wild animals [61]. These protozoa are among the main disease-causing agents of diarrhea in the world [62]. Both species form resistance structures that allow them to survive outside the host; they can thus be transmitted through water [63]. Additionally, both species have a low infective dose (ID_50_), specifically, 132 *Cryptosporidium* spp. oocysts [64] and 25–100 *Giardia lamblia* cysts [65]. Thus, a potential host consuming a low number of these microorganisms can readily develop an infection.

Based on these facts, the detection of *C. parvum* and *G. lamblia* in 50% and 44% of the Magdalena River samples, respectively (Table 4), means that the population and animals that use the water for diverse activities are exposed to parasites, which eventually could lead to an outbreak or epidemic. Children between 0 and 14 years of age and immunodeficient persons are susceptible.

For the Cuitzmala river, a rural area in Jalisco, Mexico, Tapia-Palacios (2012) [37] reported a range of 0–87 oocysts/L *C. parvum* and 0–2,655 cysts/L of *G. lamblia*, which in contrast with the results of this analysis for the urban area, though both species were detected in the two areas. The range in the natural zone was lower for both species (0–1,000 oocysts of *C. parvum* and 1,000–5,000 cysts/L of G. lamblia), which is in contrast with the urban areas that exhibited higher ranges (0–23,000 oocysts/L of *C. parvum, and* 0–28,000 cysts/L of *G. lamblia*). This can be explained by the input of wastewater from the urban area [12]. Untreated water could be increasing the number of oocysts present in the zone, leading to a potential population exposure risk because the population densities range from 1–2 log units more than the infective dose.

Because these rivers represent the only surface water source from within the Basin for Mexico City, particular concern should be held regarding the presence of certain microorganisms, such as the protozoa *Giardia* and *Cryptosporidium* and adenovirus, which are relatively resistant to the chlorination process in comparison to other microorganisms. Additionally, the presence of opportunistic and/or potentially pathogenic bacteria in a surface water source that is chlorinated and distributed to the population suggests that there should be additional funds to consider preventive measures, consistent with the practices carried out in other countries concerning water intended for human use.

Physical removal is critical to the control of *Cryptosporidium* because the organism is highly resistant to standard disinfection practices [66], [67]. As mentioned by Betancourt and Rose (2004) [68], the effectiveness of conventional disinfection by chlorination and alternative disinfection procedures, such as chlorine dioxide, ozonation and ultraviolet irradiation (UV), toward inactivating *Cryptosporidium* has been the focus of current research. Recent USEPA criteria regarding disinfection processes for water containing this pathogen suggest disinfection with ultraviolet light or ozone and/or filtration [69]. The goals for systems that treat surface water must be disinfection and filtration to remove or inactivate 99.9% of *Giardia lamblia*, 99% of *Cryptosporidium* oocysts and 99.99% of viruses [69]. In Mexico and other developing countries, modern treatment processes should be applied, given the presence of these microorganisms in the surface waters.

One of the first steps when dealing with rivers is understanding their flow and any variations that occur throughout the year and across different years. In Mexico, monitoring practices were initiated but have since been abandoned for many rivers throughout the country. A sustainable approach to water management, especially in light of climate change, would be to monitor and report environmental and social conditions to provide a guide toward a more sustainable water management strategy.

The regimes of the Magdalena and Eslava rivers belong to homonymous basins that have been modified, and both exhibit two distinct historical periods. The periods are defined by the construction of excess hydraulic infrastructure works, such as gabion dams, 50 on the Magdalena and 18 on the Eslava (Figure 1), and marginal drains. The management of the flows in the Magdalena and Eslava rivers has caused significant fluctuations in flow magnitude (69%), has increased the frequency of maximum ordinary flows and has caused a significant increase (176%) in the minimum flow magnitude. This NFR alteration will certainly have consequences for the river communities in the natural area and for system rehabilitation.

These regime changes may contribute to the observed water quality degradation and contamination of the systems because the loss of velocity along a slope and other discontinuities in the river affect the inherent auto-depuration processes. As recorded in our fieldwork, the observed higher concentrations of the physicochemical variables indicate the presence of higher levels of contaminants in the lower section of both basins, especially as the urban zone is entered.

Wastewater discharges also contribute to the degradation of water quality. Hence, even if discharge does not contribute significantly to flow volume, its effect on water quality is magnified due to the decrease in flow velocity. Further, wastewater discharge increases the concentrations of various water quality attributes.

In some rivers, contaminants are diluted through their tributaries. However, in this case, the Eslava River contributes its own contaminant load, which for some variables is orders of magnitude higher than the Magdalena's water, thereby hampering the auto-depuration process.

It is necessary to treat the residual wastewater dumped into the river and recover the fluvial processes and function of the rivers as ecosystems. This approach would provide several advantages. For example, there would be more water produced in the higher basin, which could be disinfected and distributed to the population. Additionally, a decrease in the amount of water that flows to the drainage system and that must be pumped out of the Basin of Mexico would be realized, as well as additional amounts of water once the wastewater has been treated and reused for irrigation near the river.

In this study, we demonstrate a practical and representative approach to achieving a detailed water quality program that could allow for the detection of change points. Additionally, we identify the problems at some locations and various methods for improving the respective environmental conditions. In this particular case, it is also of important that locations for the required wastewater treatment plants be suggested, as well as the type of plant and the installed capacity.

The water to be treated is considered domestic wastewater based on its Biochemical Oxygen Demand (DBO_5_) and Chemical Oxygen Demand (COD), according to Metcalf and Eddy (1996) [70].

It is important to note the need for treatment plants to improve water quality. The wastewater discharge along the river has high concentrations of nitrogen and phosphorus forms, total dissolved solids, high conductivity, and a BOD_5_ of approximately 130 mg/L to 240 mg/L (results not shown). These factors, combined with the high microorganism counts, argue in favor of the installation of several wastewater treatment plants (WWTPs) [71]. A general concept for viable plants and processing methods was presented by Orta de Velasquez (2008) [23], although treatability tests would be required, along with a specific design for each plant with an associated project feasibility study.

The wastewater treatment plants considered for this river, in all cases, included tertiary treatment. This treatment is implemented by biological secondary treatment, and the advanced treatment is implemented by physico-chemical treatment. The first treatment of activated sludge helps remove nutrients (N and P) and both soluble and colloidal biodegradable organic matter. Tertiary filtration is used to treat wastewater intended for reuse to remove any suspended matter that was not removed in the previous operations. Microbial standards can be met using both filtration (physical removal) and disinfection (inactivation). The removal and inactivation capabilities vary depending on the filtration technology or disinfectant applied. The first wastewater treatment plant constructed in the area of the Magdalena River was finished at the end of 2013, not clear if it is in operation at the moment. With the goal of removing both nutrients and pathogens, advanced treatment, which includes activated sludge, tertiary filtration and ultraviolet disinfection, was implemented. In this case, an ultraviolet light disinfection process was applied, which can effectively inactivate most viruses (111 mW*s/cm^2^ for 6-log inactivation), *Giardia* (121 mW*s/cm^2^ for 2-log inactivation) and *Cryptosporidium* (1,900 mW*s/cm^2^ for 2-log inactivation) [72]. With this plant being operational, the river must have a basic flow level along its path to preserve some system functions.

At least two WWTP are proposed. The first could be placed near sites M11 and E8, which could treat low-quality water flowing from both rivers. A second WWTP could be placed before site M19, where the treated water could be used for irrigation in one of the few large green areas of the southern part of the city, known as *Viveros de Coyoacán*. The potential locations for these two plants are presented in Figure 1.

From the perspective of the ecosystem services provided by this lotic system, the amount of water provided by the Magdalena-Eslava river system accounts for the ecological flow in the natural area, after which the water goes through a potabilization process and is distributed for human use and consumption in the southern area of the city. The amount of water that is contaminated when clean water from the natural areas of both rivers combines with untreated wastewater is not accounted for, but it is necessary to consider the financial resources that are being wasted on these relatively small rivers.

The current average flow of the Magdalena river is 0.45 m^3^/s, based on measured flow, and the cost per m^3^ with the water subsidy is $8.17 (equivalent to 0.628 USD at an exchange rate of US $1 = $12.99 Mexican peso) Mexican pesos; without subsidy, the cost is $25.97 (equivalent to US $1.99) (SACM, Official Receipt. *Derechos por el suministro de agua. Magdalena Contreras. Manzana Baja*. September, 2013) [73]. Therefore, the amount of water lost accounts for 14,191,200 m^3^ per year, which means a loss of $115,942,104 Mexican pesos (equivalent to US $8,925,489) at the subsidized cost, or $368,545,464 Mexican pesos (equivalent to US $28,371,475). In this example, we have only considered the Magdalena River, which has better water quality and more abundant flow. An equivalent of this at Mexico City prices for bottled water at $5.66 per liter ($5,660 per m^3^) is an estimated $79,470,720,000 Mexican pesos (equivalent to US $6,117,838,337).

As part of any megacity water management strategy, there is a need to consider the amount of water lost and the financial capital that those losses represent. There is an urgent need to rehabilitate and recover the flow of the river and its water quality, which is an approach that could be replicated in more than 40 other rivers around Mexico City. The ecosystem service of clean water is being lost in the megacity, where water must be pumped at a very high cost from either groundwater systems or distant basins. These rivers serve as one example of similar situations that have arisen for other rivers in the Basin of Mexico, where water could be managed in a more sustainable way in the vicinity of this megacity. Doing so would yield enormous environmental and economic benefits in the long term.

From the perspective of providing water, water quality represents a fundamental issue. Better use of the existing water sources can be achieved in the MCMA. The rehabilitation of the Magdalena-Eslava river system could provide an example for other local and remote locations to follow.

Health issues are at stake, so action must be taken to ensure water quality in this particular system. The contaminated water flowing through the urban area should be improved to avoid future deleterious effects to the population.

Considering the treatment of wastewater that flows through the urban area, this water could also be used for irrigation in the green areas in the southern area of Mexico City.

## Conclusions

The flow regimes of the Magdalena and Eslava rivers have been modified by the increase of hydraulic infrastructure constructed along the river, resulting in decreased maximum flows and increased minimum flows. These changes in flow regimes are directly related to the quality of the river water, due to the loss of velocity in the flow and the loss of continuity of flow.

The water quality of this system deteriorates as the river flows from a natural forested area to a semi-urban area, passing irregular settlements without basic water and drainage services before flowing then into an urbanized area.

The water quality is worst in the Eslava River at the confluence point, M12, after which either a dilution effect or an artifact of the complex drainage system arises, leading to an apparent improvement in the wastewater quality downstream. Organic matter, such as that found in the discharge, affects the oxygen balance and is usually accompanied by microbiological contamination.

The domestic and agricultural wastes contribute phosphates and ammonia nitrogen. Agriculture also contributes to the dissolved solid levels measured in the river.

The Magdalena Eslava river system could be used more efficiently. The river receives domestic discharge, and a portion of the flow passes through the *Delegación Magdalena Contreras*, where no industry is present.

The construction of at least two wastewater treatment plants is proposed, with the goal of restoring the river's water quality and maintaining the flow in the system. The suggested placement of these treatment plants is based on the low flow that occurs during the dry season and the sewer system conditions. By considering the landscape design to be a linear park, the treated wastewater flow was defined to rely on water flow and high water quality for recreational use. Additionally, the potential to reuse this water for green area irrigation and car washing exists. Treating the wastewater in this area of Mexico City would be relatively simple in comparison to treating the wastewater in other industrial areas of this metropolitan area.

Human activities, which have increased significantly in recent decades, have impacted the environment. The scale of urbanization, industrial operation and agricultural production has reached a point at which these activities impact the quality of the hydraulic resources. This is a global phenomenon to which Mexico City is not an exception.

Wastewater in the cities of the developing world transports a combination of liquid and solid residues from regular and irregular residential areas, public institutions, and industrial and commercial establishments. These residues eventually reach pluvial, surface and groundwater. Due to the diversity of compounds and microorganisms in wastewater, such as those observed in this study, it is not possible to provide details for all compounds but only to give an idea of the water technology required for improving water quality, with a view toward reuse and sustainability. Undoubtedly, there exist other compounds that were not measured as part of this evaluation, such as endocrine disruptors or organic compounds; these compounds must be evaluated, especially in the urban section of the lotic system, to develop a complete picture.

The amounts of several types of microorganisms in the urban zone, each with a different resistance to water and wastewater treatment, must be considered from the public health perspective because these microorganisms represent an important exposure factor for the local population.

Mexico City has lost the majority of its rivers due to mismanagement. The case of the Magdalena and Eslava rivers represents an opportunity to rehabilitate a major river system and could serve as a model for other waterways.
